# Pulsed Erlotinib as Sole Treatment of Leptomeningeal Carcinomatosis: Can We Avoid the Use of Intrathecal Therapy?

**DOI:** 10.14740/wjon859w

**Published:** 2014-12-03

**Authors:** Cheruppolil R. Santhosh-Kumar, Deborah Gray, Dave J. Fisher

**Affiliations:** a Vince Lombardi Cancer Clinic, Aurora Cancer Care, Aurora Health Care, Sheboygan, WI, USA

**Keywords:** Leptomeningeal, EGFR, Erlotinib, Adenocarcinoma, Non-small cell

## Abstract

Leptomeningeal carcinomatosis, a not uncommon complication of non-small cell lung cancer, is associated with poor prognosis, and median survival is reported in case series as weeks to months. The advent of targeted therapy may have positively impacted the prognosis of such patients recently. Standard approaches to treatment of leptomeningeal metastasis include intrathecal chemotherapy with or without cranial or craniospinal radiation and additional systemic therapy. We report a case of leptomeningeal metastasis in epidermal growth factor receptor overexpressing lung adenocarcinoma showing an excellent response with pulsed doses of erlotinib as the only therapy.

## Introduction

Leptomeningeal carcinomatosis (LC) is a not uncommon complication of non-small cell lung cancer [1]. Generally, LC is associated with poor prognosis, and median survival is reported in case series as weeks to months [2-5]. However, the advent of targeted therapy may have positively impacted the prognosis of such patients recently [4]. Intrathecal chemotherapy is the mainstay of treatment for LC. In addition, cranial or craniospinal radiation and systemic therapy are often used [5].

We report a case of leptomeningeal metastasis in epidermal growth factor receptor (EGFR) overexpressing lung adenocarcinoma showing an excellent response with pulsed doses of erlotinib as the only therapy.

## Case Report

A 64-year-old white woman presented with neck swelling, shortness of breath and neck pain in November 2011. Three months prior, she had been diagnosed with an unprovoked deep venous thrombosis of the left leg and thigh and initiated on warfarin for anticoagulation. The patient worked in a chair factory. She had quit her 30-year habit of smoking one pack a day in 1994.

Medical history was significant for deep venous thrombosis and pulmonary embolism in the postpartum period of her fourth pregnancy in 1971. Family history was significant as both the patient’s mother and a daughter died of breast cancer, the latter with LC. There was ovarian cancer, brain cancer, lung cancer and non-Hodgkin lymphoma in her extended family. Physical examination was remarkable only for mild edema of the left arm.

A computed tomography (CT) scan of the neck and chest showed thrombosed right internal jugular and brachiocephalic veins. A focal area of consolidation was located in the medial segment of the right middle lobe. There were segmental pulmonary emboli and a mild right pleural effusion. Pleural fluid analysis and biopsy of the right scapular lesion showed malignant cells consistent with adenocarcinoma. An EGFR mutation, exon 19 deletion, was detected in the pleural fluid sample. There was no evidence of a T790M or other mutation.

**Figure 1:**
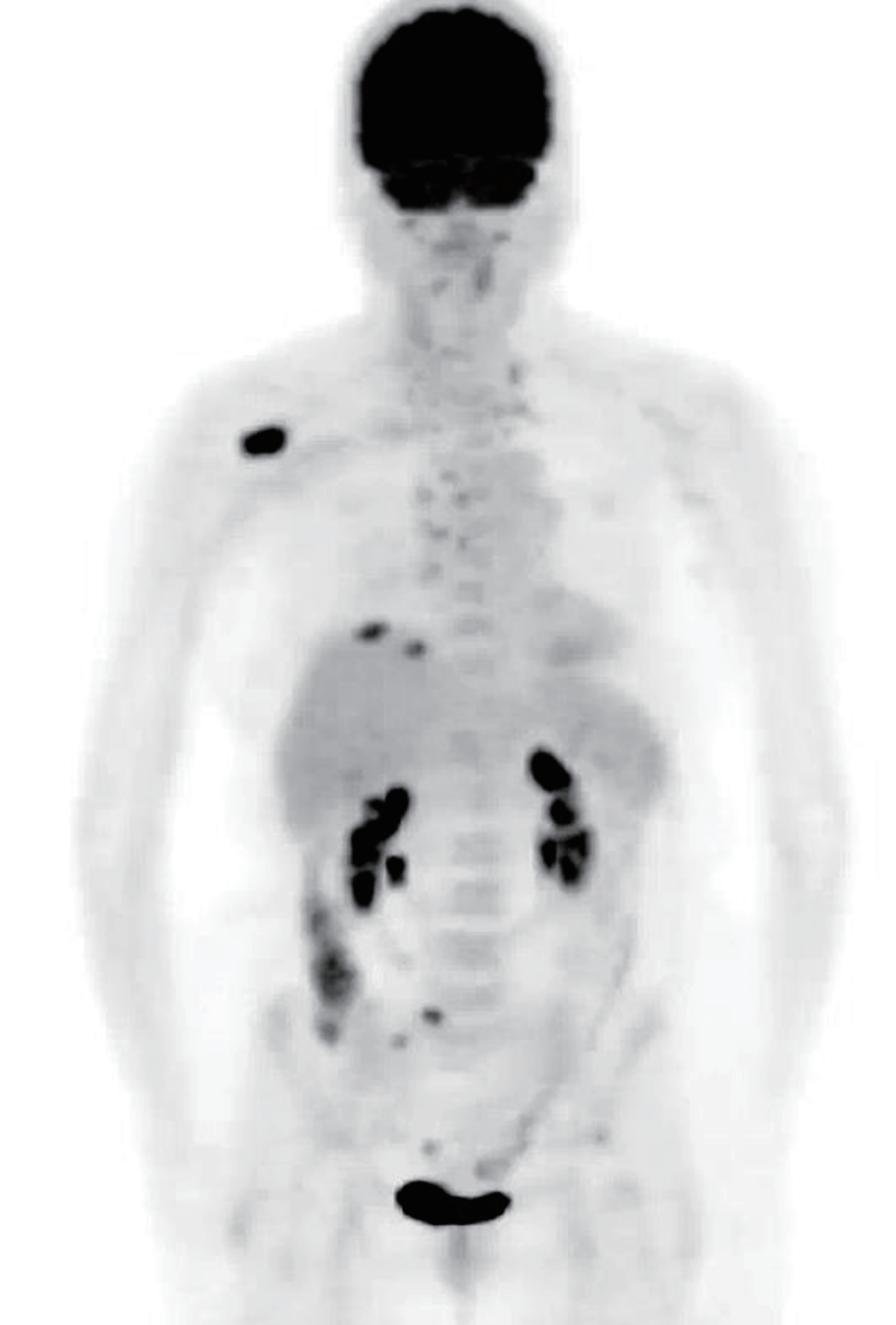
Computed tomography-positron emission tomography performed prior to treatment. Maximum intensity projection image shows mass within right lung base. Adenopathy with abnormal uptake is present throughout the mediastinum and neck. Largest bone metastasis involves right scapula.

A CT-positron emission tomography (PET) scan (Fig. 1) showed a 2.3-cm mass in the anterior right lung showing increased metabolic activity with a standardized uptake value (SUV) of 3.8. Multiple metabolically active lymph nodes were located in the mediastinum, left supraclavicular region and retroperitoneum. Multiple bone metastases were detected in the ribs, sacrum and the right scapula. A right scapular lesion had an SUV of 10.6. Magnetic resonance imaging (MRI) of the brain did not show any evidence of parenchymal or leptomeningeal metastatic disease.

The patient started oral erlotinib, 150 mg daily, and subcutaneous denosumab, 120 mg monthly, in addition to daily subcutaneous low molecular weight heparin. The patient rapidly became asymptomatic and significant decrease in the lung mass, improvement of pleural effusion and resolution of pulmonary emboli were noted on chest CT 3 months after diagnosis.

**Figure 2:**
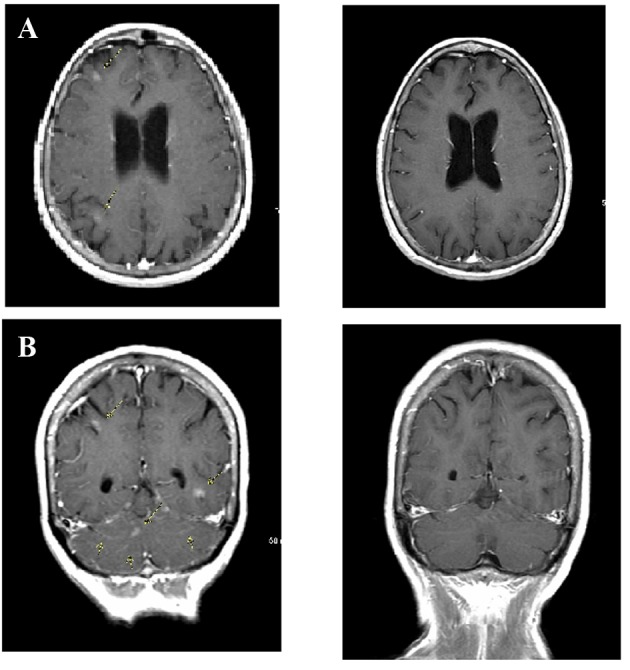
Leptomeningeal carcinomatosis. (A) Brain magnetic resonance imaging (MRI) obtained when patient first presented with neurologic deficits shows multiple sites of leptomeningeal enhancement on post-contrast T1-weighted sequences (arrows). (B) Following pulsed-dose erlotinib therapy, repeat MRI scan showed complete resolution of tumor.

In July 2013, 18 months after diagnosis, the patient began experiencing episodes of confusion, headaches, bizarre sensations in the head and difficulties with short-term memory. An MRI of the brain showed patchy areas of leptomeningeal enhancement (Fig. 2A). A cerebrospinal fluid (CSF) study showed mild decrease in CSF glucose, normal protein level and the presence of multiple mononuclear cells consistent with adenocarcinoma. A CT-PET scan showed evidence of response to therapy with decrease in size of the lung mass to 6 mm without increased metabolic activity, resolution of pleural effusion and sclerosis of bone metastasis without increased metabolic activity.

A diagnosis of LC was made and treatment options including intrathecal chemotherapy and cranial or craniospinal radiation were discussed. The patient refused these treatments mainly because of the death of her daughter who had LC related to an Ommaya reservoir for treatment of breast cancer several years ago. The patient was placed on pulsed erlotinib therapy with dose adjustments depending on toxicity. The patient derived her own optimal dosage of 600 to 750 mg daily for 3 days on and 2 days off resulting in a total dose of 2,550 mg per week. The patient noticed complete resolution of neurological symptoms in about a week and has remained symptom free at the time of this report more than 12 months after diagnosis of LC. The patient is symptomatic only in terms of grade 1 dermatologic toxicity and grade 1 diarrhea secondary to erlotinib. Results of spinal fluid analysis continue to show adenocarcinoma cells; however, multiple CSF samples failed to provide enough cells for molecular analysis.

CT-PET scan in March 2014 did not show any evidence of increased metabolic activity. Craniospinal MRI in April 2014 (Fig. 2B) did not show any evidence of metastatic disease or leptomeningeal enhancement.

## Discussion

Prolonged survival with leptomeningeal metastasis without intrathecal therapy or radiation therapy is uncommon in lung cancer. In two recent reports of patients with non-small cell lung cancer and leptomeningeal metastasis, the majority of whom were treated with intrathecal chemotherapy, the median survival was 3 months [2-4].

Our patient’s reluctance to undergo radiation therapy or intrathecal treatment led to the investigation of pulsed erlotinib, and has resulted in a major response systemically and stability of the disease in the central nervous system.

Prior reports of high-dose pulsatile erlotinib (weekly doses of 1,500 mg) combined with other treatment, including intrathecal therapy, in EGFR overexpressing non-small cell lung cancer patients with LC showed a better median survival of 12 months [5]. Pharmacokinetic studies have shown that high doses of oral erlotinib can result in higher CSF penetration [6]. It is possible that the higher weekly dose of 2,550 mg may have contributed to the prolonged response in our patient.

Similar to non-small cell lung cancer, prolonged survival has been reported in patients with HER2-positive breast cancer and brain and leptomeningeal metastasis undergoing anti-HER2 therapy [7]. High-dose intravenous methotrexate therapy has been found to be comparable to intrathecal methotrexate in patients with lymphomas and leukemias [8, 9]. Intrathecal therapy could be associated with significant complications [10]. It is possible that we may be able to avoid the morbidity associated with intrathecal chemotherapy and craniospinal irradiation with the use of systemic biological therapy in patients with leptomeningeal metastasis where effective biologic therapy is available.

A clinical trial comparing biological therapy to current standard of care (intrathecal therapy or radiation) is planned for patients with leptomeningeal metastasis and targetable mutations in lung and other cancers.
